# Instruments to assess adherence to medication in people living with HIV: a scoping review

**DOI:** 10.11606/s1518-8787.2022056004475

**Published:** 2022-11-18

**Authors:** André Pereira dos Santos, Jéssica Fernanda Corrêa Cordeiro, Isabela Fernanda Larios Fracarolli, Euripedes Barsanulfo Gonçalves Gomide, Denise de Andrade

**Affiliations:** I Universidade de São Paulo Escola de Enfermagem de Ribeirão Preto Ribeirão Preto SP Brasil Universidade de São Paulo. Escola de Enfermagem de Ribeirão Preto. Ribeirão Preto, SP, Brasil; II Universidade de São Paulo Escola de Educação Física e Esporte de Ribeirão Preto Ribeirão Preto SP Brasil Universidade de São Paulo. Escola de Educação Física e Esporte de Ribeirão Preto. Grupo de Estudos e Pesquisa em Antropometria, Treinamento e Esporte. Ribeirão Preto, SP, Brasil; III Universidade de São Paulo Escola de Educação Física e Esporte de Ribeirão Preto Ribeirão Preto SP Brasil Universidade de São Paulo. Escola de Educação Física e Esporte de Ribeirão Preto. Ribeirão Preto, SP, Brasil; IV Human Exposome and Infectious Diseases Network Ribeirão Preto SP Brasil Human Exposome and Infectious Diseases Network. Ribeirão Preto, SP, Brasil; V Claretiano – Centro Universitário Batatais SP Brasil Claretiano – Centro Universitário. Batatais, SP, Brasil

**Keywords:** HIV Infections, therapy, Medication Adherence, Treatment Refusal, HIV Long-Term Survivors, Review

## Abstract

**OBJECTIVE:**

To compile the instruments validated in Brazil for assessing adherence of people living with HIV to antiretroviral therapy.

**METHODS:**

Scoping review using the Web of Science, Scopus, Medline (via PubMed), Embase, BDENF, CINAHL and Lilacs databases. In addition, the Preprints bioRxiv, Google Scholar and OpenGrey servers were checked. There was no language restriction for the search, and it considered articles published from the year 1996 onwards.

**RESULTS:**

Three publications were included in the qualitative synthesis. Following were the instruments identified “*Questionário para Avaliação da Adesão ao Tratamento Antirretroviral*” (Questionnaire for Assessment of Adherence to Antiretroviral Treatment) developed in Porto Alegre (RS) and published in 2007; the “*Escala de autoeficácia para adesão ao tratamento antirretroviral em crianças e adolescentes com HIV/Aids*” (Self-efficacy Scale for Adherence to Antiretroviral Treatment in Children and Adolescents with HIV/Aids) developed in São Paulo (SP) and published in 2008; and the “*WebAd-Q, um instrumento de autorrelato para monitorar a adesão à terapia antirretroviral em serviços de HIV/Aids no Brasil*” (WebAd-Q, a self-report instrument to monitor adherence to antiretroviral therapy in HIV/Aids services in Brazil) developed in São Bernardo do Campo (SP) and published in 2018. The instruments were validated in Brazil, and presented statistically acceptable values for psychometric qualities.

**CONCLUSION:**

The instruments to assess adherence of people living with HIV to antiretroviral therapy are validated strategies for the Brazilian context. However, their (re)use in different settings and contexts of the nation should be expanded. The use of these instruments by health professionals can improve the understanding of factors that act negatively and positively on antiretroviral therapy adherence, and the proposition of strategies intended to consolidate good adherence and intervene in the treatment of people with low therapeutic engagement.

## INTRODUCTION

With the advent of antiretroviral therapy (ART) after 1987 in some parts of the world, and in Brazil since 1996, with public, free, and universal access, advances in the constitution and strategy of administration of antiretroviral drugs transformed the understanding about human immunodeficiency virus (HIV) infection worldwide, whether or not associated with acquired immunodeficiency syndrome (Aids), changing the focus from a fatal disease to a chronic infection^1,2^.

Even with the prolonged remission of HIV described in literature^3,4^, to date there is no cure, and the treatment that brings the viral load to an undetectable level (< 40 copies/ml) has as its main component the patient’s adherence to antiretroviral therapy^5^. HIV control and health maintenance are promoted by the continuous and appropriate use of the ART, whose effectiveness is linked to the intake of drugs as prescribed, determining adherence to treatment. For satisfactory adherence, the use of antiretroviral drugs should be as close as possible to the prescription provided by the healthcare team, in order to comply with schedules, doses, and other instructions^6^. High levels of adherence have been consistently associated with better virologic, immunological, and clinical outcomes, with consequent increases in survival and quality of life for people living with HIV^8^. Additionally, considering that one of the pillars of the HIV epidemic control programs around the world is the strategy called U = U (undetectable = untransmissible), adherence to therapy has a positive impact on reducing the number of bound partner transmissions^9,10^. On the other hand, drug treatment abandonment or incorrect adherence can facilitate infection by opportunistic diseases and lead to death. In short, non-adherence to antiretroviral therapy negatively impacts social and political aspects, both for the public investment made and for controlling the epidemic^11^.

In recent years, Brazil has seen a decrease in the number of HIV infections. Between 2007 and 2020, 342,459 cases of HIV infection were reported to the *Sistema de Informação de Agravos de Notificação* (Brazilian Case Registry Information System)^11^. The Unified Health System has sustained its actions to ensure diagnosis and treatment for HIV, even during the covid-19 pandemic. The Ministry of Health prepared a “*Monitoramento durante a pandemia da covid-19 – dados relacionados ao HIV*” (Monitoring during the covid-19 pandemic - HIV-related data) panel, in which it brought important information about the numbers of people who started antiretroviral therapy each year. For example, in 2019, a total of 68,482 patients adhered to the ART; the following year, in 2020, there were 55,180 new adhesions^12^.

The barriers and facilitators for adherence to antiretroviral therapy are multifactorial, involving social, economic, systemic/professional (healthcare teams) issues, side effects to treatment, to the disease, and the uniqueness of the person living with HIV^13^. Each country and their regions may have specificities that could be configured as barriers to treatment; for example, countries such as Uganda, Tanzania, and Botswana may present obstacles in which hunger, waiting time for medication, and transportation costs are strong complicators for adhering to the ART^14^. In Brazil, the main factors associated with non-adherence to therapy are sociodemographic aspects, such as age (young), marital status (single), self-reported skin color (non-white), education (low level), income (low socioeconomic status), and vulnerability to HIV/Aids, such as licit and illicit drug use, use of health services (non-adherence to visits and contact with more than one healthcare service), added with clinical laboratory follow-up (perception of side effects and complexity of the therapeutic regimen)^15,16^.

Due to the different measurement instruments adopted at regional level, there is no national estimate of adherence to antiretroviral therapy; however, a recent literature review found that this rate rages between 18% and 74.3% depending on the location^17^.

Among the different instruments and techniques used to assess adherence of people living with HIV to therapy are the antiretroviral dispensation record^18^, medical record analysis^19^, self-completion questionnaire^20^, simplified questionnaire for assessment of ART adherence-SMAQ21^21^, etc. It has been observed, however, that the proposals for this assessment in healthcare services in Brazil do not use validated instruments and techniques^17^, which can lead to errors in verification and interpretation of results^22^.

This study aimed at tracking the instruments validated in Brazil to assess adherence of people living with HIV to antiretroviral therapy.

## METHODS

### Study Design

A scoping review is intended to address broad issues, focusing on more comprehensive results. This type of review rigorously and effectively identifies, examines, and systematizes a concept or unique trait of each study by identifying the nature of a broad field of knowledge^23,24^. The scoping review is systematized, and some steps are required to conduct it. Among these steps, five stages are considered mandatory and one optional: (1) identification of the research question; (2) identification of relevant studies; (3) selection of studies; (4) data mapping; (5) pooling, analysis, and summary of data; and (6) consultation with researchers (optional)^23,24^. This scoping review followed the guidelines of the checklist for systematic reviews and extension of meta-analyses for scoping reviews (Prisma-ScR)^25^.

### Definition of the Question

In determining the research question, we decided to use the population (P), concept (C) and context (C)^26^ strategy. Here, (P) = people living with HIV who take antiretroviral therapy, (C) = instruments on adherence to antiretroviral therapy, and (C) = instruments validated in Brazil. The guiding question of this study was: Are there validated instruments in Brazil to assess adherence to antiretroviral therapy in people living with HIV?

### Period

The search for studies was conducted in December 2020 by two researchers independently, avoiding bias in the number of articles.

### Data Collection

Major healthcare databases were selected for search: Web of Science (WOS/ISI), Scopus, Medical Literature Analysis and Retrieval Online (Medline/PubMed), Embase, BDENF, The Cumulative Index to Nursing and Allied Health Literature (CINAHL), and *Literatura Latino-Americana e do Caribe em Ciências da Saúde* (Lilacs). In addition, the Preprints bioRxiv, Google Scholar and OpenGrey servers were checked for their recognition in the academic community, and due to the expressive number of documents available.

### Selection Criteria

This search included primary studies, descriptive studies, reviews, editorials, and manuals published from 1996 onwards (the beginning of antiretroviral therapy implementation in Brazil as a universal and public access policy against HIV), without language restriction. The available full texts that answered the research question were selected. Articles repeated in more than one data source were counted only once. All studies found in the search were imported into Rayyan^®^ software, where the entire analysis and selection process was performed.

### Instrument Used to Collect Information

The descriptors used in this research were selected through the “*Descritores em Ciências da Saúde*” (DeCS) and “Medical Subject Headings” (MESH) databases, which are highlighted in Chart 1.

**Chart 1:** Search strategies used on the databases and data repositories.

Database	Search Strategy
PubMed/Medline	(“Acquired Immunodeficiency Syndrome” OR HIV) AND (“medication adherence” OR compliance) AND (“Surveys and Questionnaires”)
CINAHL	(“Human Immunodeficiency Virus” OR “Anti-HIV Agents”) AND (“Medication Compliance”) AND (“Structured Questionnaires” OR “Surveys”)
Web of Science	(Acquired Immunodeficiency Syndrome OR HIV) AND (medication adherence OR compliance) AND (Surveys and Questionnaires)
Scopus	(“Acquired Immunodeficiency Syndrome” OR HIV) AND (“medication adherence” OR compliance) AND (“Surveys and Questionnaires”)
Embase	Acquired Immunodeficiency Syndrome AND medication compliance AND questionnaire
BVS (Lilacs, BDENF)	(Acquired Immunodeficiency Syndrome OR HIV) AND (medication adherence OR compliance) AND (Surveys and Questionnaires)
	(Síndrome de inmunodeficiencia adquirida O VIH) Y (adherencia o cumplimiento a la medicación) Y (Encuestas y cuestionarios)
BioRxiv	“Acquired Immunodeficiency Syndrome AND medication adherence AND Surveys and Questionnaires”
Google Scholar	“Acquired Immunodeficiency Syndrome AND medication adherence AND Surveys and Questionnaires”
OpenGrey	“Acquired Immunodeficiency Syndrome AND medication adherence AND Surveys and Questionnaires”

### Data Processing and Analysis

Next, the data extracted by two independent reviewers were pooled, analyzed, and summarized. Questions and inconsistencies were analyzed and discussed by a third reviewer. Data were recorded in Microsoft Office Excel^®^ spreadsheet, version 2010, and presented as to identification (author and year), objective, method, main results, biases and limitations of the study^27^.

## RESULTS

The search in scientific literature for these validated instruments that assess patient adherence to antiretroviral therapy in Brazil resulted in the inclusion of three studies. The Figure shows the studies’ search, identification, exclusion, and selection process, according to the Prisma recommendations.

**Figure:**
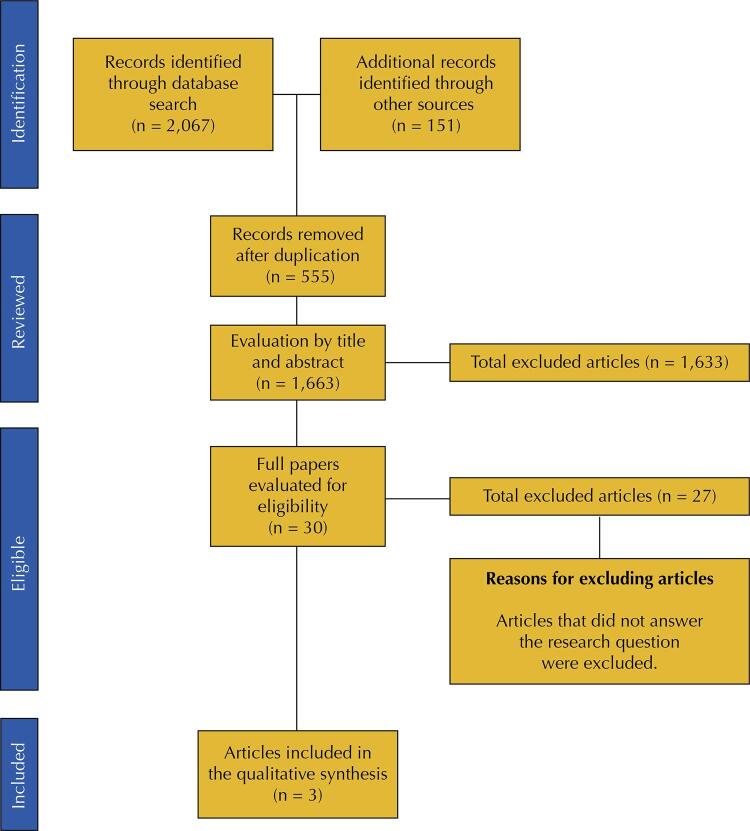
Flowchart of the selection of studies retrieved from the databases, adapted from the Preferred Reporting Items for Systematic Review and Meta-Analyses (Prisma) on the validated instruments that assess adherence to antiretroviral therapy in people living with HIV in Brazil.

Following were the instruments identified “*Questionário para Avaliação da Adesão ao Tratamento Antirretroviral*” (Questionnaire for Assessment of Adherence to Antiretroviral Treatment) developed in Porto Alegre and published in 2007^28^; the “*Escala de autoeficácia para adesão ao tratamento antirretroviral em crianças e adolescentes com HIV/Aids*” (Self-efficacy Scale for Adherence to Antiretroviral Treatment in Children and Adolescents with HIV/Aids) developed in São Paulo and published in 2008^29^; and the “*WebAd-Q, um instrumento de autorrelato para monitorar a adesão à terapia antirretroviral em serviços de HIV/Aids no Brasil*” (WebAd-Q, a self-report instrument to monitor adherence to antiretroviral therapy in HIV/Aids services in Brazil) developed in São Bernardo do Campo and published in 2018^30^. All publications included in the qualitative synthesis of this scoping review were written in Portuguese and English, except the publication by Remor; Milner-Moskovics; Preussler, 2007^28^, published exclusively in Portuguese. The three publications included were found in the Scopus database. The information extracted from these studies on identification, objective, method, main results and biases/limitations are presented on Chart 2.

**Chart 2:** Summary of studies included in the scoping review regarding the question: “Are there validated instruments in Brazil to assess adherence to antiretroviral therapy in people living with HIV?”

Identification (author/year)	Objective	Method	Main results	Study limitations
Remor E, Milner-Moskovics J, Preussler G., 2007^28^	Translate, adapt and validate the questionnaire for use in Brazil: *Cuestionario para la Evaluación de la Adhesión al Tratamiento Antiretroviral (CEAT-VIH*)”. It is a self-administered instrument to identify the degree of adherence to antiretroviral treatment in patients with HIV infection.	This methodological study was based on a questionnaire translated from the original Spanish into Portuguese, with a back translation process (Spanish/Portuguese/Spanish), and verbal evaluation of understanding with a small group of patients. The analysis of psychometric properties involved 59 patients on antiretroviral treatment from Porto Alegre, Rio Grande do Sul, Brazil. Internal consistency and external criterion-related validity, sensitivity and specificity supported validation. For the final version of the CEAT-VIH (20 questions), the total score is obtained by the sum of all items (minimum possible value 17, maximum possible value 89).	Adequate reliability of the questionnaire (α = 0.64) and validity related to an external criterion (viral load; r = -0.48; p < 0.001) were observed. Also, adequate sensitivity (79.2%) and specificity (57.1%) of the questionnaire were observed for detection between individuals with undetectable and detectable viral load. The optimal cutoff point suggested by the analysis is ≥ 76. The values below indicate insufficient adherence to treatment, and association with a detectable viral load. This score is associated with a sensitivity of 79.2%, and specificity of 57.1%.	1) Calculation for sample size is not presented. 2) Study in only one institution, making generalization difficult; therefore, it is not a nationally representative study. 3) The established cutoff point should be used with caution as the questionnaire is a self-administered instrument. 4) Hawthorne’s effect.
Costa LS, Latorre M do RD de O, Silva MH da, Bertolini DV, Machado DM, Pimentel SR, et al., 2008^29^	Validate a self-efficacy scale for adherence to antiretroviral treatment in children and adolescents with HIV/Aids, taking into account the perspective of parents/guardians, and evaluate its reproducibility.	Methodological study conducted at the *Hospital-Dia do Centro de Referência e Treinamento em DST/Aids* (Day Hospital of the STD/Aids Reference and Training Center) in São Paulo. We interviewed the parents/guardians of 54 children and adolescents of 6 months to 20 years of age who went through routine consultation at the service. The self-efficacy data of adherence to antiretroviral prescription was calculated in two ways: factor analysis, and pre-defined formula. The internal consistency of the scale was verified by Cronbach’s α coefficient. Validity was assessed by comparing the mean scores between groups of patients adherent and non-adherent to antiretroviral treatment (Mann-Whitney test), and calculation of Spearman’s correlation coefficient between scores and clinical parameters. Reproducibility was verified using the Wilcoxon test, the intraclass correlation coefficient, and Bland-Altman plots.	The self-efficacy scale consists of 21 questions. It showed good internal consistency (α = 0.87), and good reproducibility (ICC = 0.69 and ICC = 0.75). As for validity, the self-efficacy scale for following antiretroviral prescription was able to discriminate patients with adherence and with insufficient adherence to antiretroviral treatment (p = 0.002), and showed significant correlation with CD4 count (r = 0.28; p = 0.04). Results are added with the fact that children/adolescents with adherence to antiretroviral treatment have higher expectancies of self-efficacy than those with insufficient adherence. The self-efficacy scale for following antiretroviral prescription can be used to assess adherence to antiretroviral therapy in children and adolescents with HIV/ Aids, taking into account the perspective of parents/caregivers.	This is not a Nationally representative study. Parents/caregivers responded for the children/adolescents. The perception of the target population of the study was not considered. Hawthorne’s effect.
Vale FC, Santa-Helena ET de, Santos MA, Carvalho WM do ES, Menezes PR, Basso CR, et al., 2018^30^	Present the development and validation of the WebAd-Q Questionnaire, a self-report instrument to monitor adherence to antiretroviral therapy in HIV/Aids services in Brazil.	Methodological study. The WebAd-Q is an electronic questionnaire that contains three questions about taking antiretroviral drugs in the last week. It was constructed based on interviews and focus groups with 38 patients. Validity was verified in a study with a sample of 90 patients older than 18 years, on antiretroviral therapy for at least three months. The following comparative adherence measures were used: electronic monitoring, pill count, and self-report interview. The WebAd-Q was completed on day 6 twice, at least 1 hour apart. The viral load of patients was obtained from service records. Agreement between WebAd-Q responses, associations and correlations with viral load and performance against other measures of adherence were analyzed.	Among guest patients, 74 (82.2%) responded to the WebAd-Q. No difficulties in answering the questionnaire were reported. The average response time was 5 min 47 sec. The set of the three WebAd-Q questions obtained 89.8% agreement, with a Kappa of 0.77 (95% CI 0.61-0.94). WebAd-Q insufficient adherence responses were associated with detectable viral load. Moderate correlations of viral load with insufficient adherence scale according to WebAd-Q were obtained. For all the three WebAd-Q questions, patients with responses of insufficient adherence were also noted as less adherent according to the other measures of adherence. The WebAd-Q met the main requirements for questionnaire validation, showed high participant comprehension and association with viral load, and obtained agreement and good performance compared to competing measures.	This is not a Nationally representative study. Sample loss may have reduced the statistical power of the study. Hawthorne’s effect.

In summary, all studies included in this review validated their instruments, indicating statistically acceptable values regarding the psychometric qualities of each instrument. The main bias/limitation observed regards sample specificity, i.e., they suggest caution in using the instruments when verifying adherence to therapy by people from other regions of Brazil.

## DISCUSSION

This scoping review was driven by the mapping of instruments to measure adherence to antiretroviral therapy by people living with HIV validated in Brazil. To the best of our knowledge, this is the first study that has gathered the strategies that could be used in Brazil. The identification of these instruments is the main finding of our study, and may guide the production of relevant knowledge for health service planning and management. This review also advances the field of adherence to ART by analyzing and compiling the instruments that, based on the results achieved, can be accurately and safely interpreted, in light of the psychometric rigor of each proposal.

Considering the positive impact on the quality of life of people living with HIV who present adequate adherence to antiretroviral therapy, as well as the harms of irregularity or discontinuation of treatment, it is important to highlight that adherence is a collaborative process linked to the principle of autonomy, which implies active participation of the patient in healthcare. This cooperation between the person living with HIV and the multiprofessional team promotes the acceptance and incorporation of the therapeutic scheme into the daily treatment routine^31^.

In studies involving public health in Brazil, the instruments presented in this review^28^may improve understanding of the prevalence, incidence, and risk factors for poor adherence to ART, as well as propose strategies to improve drug adherence. In addition, the use of validated instruments will contribute to health professionals’ interventions in healthcare and better outcomes on the overall well-being of people living with HIV. The questions in the instruments included in our review include the five determinants that act negatively or positively on long-term treatment adherence established by the World Health Organization: social and economic factors, health system/team, treatment, disease, and patient-related factors^13^.

The “*Cuestionario para la Evaluación de la Adhesión al Tratamiento Antirretroviral*” (CEAT-VIH), built in 2002^32^, is a simplified instrument comprising 20 questions applicable to adults living with HIV. In addition to its validation in Brazil^28^, the CEAT-HIV has been validated in Canada^33^, Portugal^34^ and Peru^35^, suggesting its usefulness and validity to assess adherence to antiretroviral therapy in different contexts. In the validation study in Brazil, a significant and positive association was observed between the number of pills and viral load (r = 0.32; p = 0.01), suggesting that the number of pills of the therapy may be a hindering aspect for treatment adherence. Administration of a number ≤ 10 tablets is associated with better adherence. A significant inverse association was also observed between the degree of adherence to antiretroviral therapy and viral load (r = -0.48; p = 0.000). The optimal cutoff point suggested by the analysis is ≥ 76. The values below indicate insufficient adherence to treatment, and association with a detectable viral load. Viral load is the clinical indicator most adopted in studies evaluating the accuracy of instruments to assess adherence to ART, indicating the relevance of drug adherence for a better prognosis of disease recovery^17,36^. Most items of the CEAT-VIH met the expected quality criteria, and there were no lost values. It suggests that all questions were understood by patients and could be answered.

Some national studies used the CEAT-VIH, such as the one developed in the city of Fortaleza (CE)^37^, another in the mid-western region of the state of Rio Grande do Sul^38^, and in five specialized healthcare services in the state of Pernambuco^39^. Low adherence to antiretroviral therapy was observed in these different contexts. This reality that legitimizes the use of the CEAT-HIV as a tool to be used by health professionals in planning healthcare and intervention in situations that interfere with adherence.

To date, the study on validation and reproducibility of a self-efficacy scale for adherence to ART in parents or caregivers of children and adolescents living with HIV/Aids is the first in the Brazilian context. The good internal consistency of the scale in the total sample of children and adolescents (α = 0.87) showed high association between the scale items, suggesting that it is reliable to measure self-efficacy in children and adolescents with HIV/Aids, considering the perspective of parents/caregivers. It was also observed that children/adolescents adherent to antiretroviral therapy have higher expectancies of self-efficacy than those with insufficient adherence. This indicates that higher self-efficacy is associated with better TCD 4+ lymphocyte responses (r = 0.28; p = 0.040). The article on validation suggests that self-efficacy score 2 be used in clinical routine, as it is calculated by a predefined and easily applicable formula. On the other hand, score 1 - also presented in the article - is a score made up of the questions that most correlate with the questions in the scale, requiring the researcher to be familiar with factor analysis, in addition to the use of a statistical package.

Some national studies have used score 2 of the self-efficacy scale to assess adherence of children and adolescents to antiretroviral therapy, among them a study conducted in Brasilia-DF^40^ and another in the city of São Paulo^41^. The study published in 2010^40^ observed difficulties related to taking medication outside the home environment, missed doses, and delays in taking the medication. In the study published in 2012^41^, a high rate of adherence to ART was identified, regardless of the sociodemographic profile of parents/caregivers. The self-efficacy scale to assess adherence of children and adolescents to drug therapy consists of 21 questions. This scope of information allows greater detailing of the aspects that hinder non-adherence, as well as those that facilitate therapeutic adherence.

The WebAd-Q instrument is a self-administered questionnaire developed in services targeted to people living with HIV. In the development and validation study^30^ a high level of test-retest agreement and good agreement between the responses and other indicators of adherence were observed. Similar to the two other instruments included in this scoping review^28,29^, the scale of insufficient adherence obtained by WebAd-Q showed moderate association when compared to the clinical outcome (viral load), i.e., the lower the adherence to antiretroviral therapy, the higher the viral load found. The WebAd-Q considers only the patient dimension within the five determinants associated with adherence to long-term treatment established by the World Health Organization^13^. It assesses whether all medications were taken at the prescribed times and doses during the last week, with good reliability.

The WebAd-Q was used to assess adherence to ART in a non-representative sample survey conducted in the five regions of Brazil^42^. A high proportion of non-adherence to antiretroviral therapy was observed. According to the authors, the WebAd-Q is a tool that can be quickly applied, favoring the routine care to people living with HIV, allowing the identification of gaps in the provision of counseling and guidance on therapy, and also allows the development of innovative strategies to prevent non-adherence.

We highlight the following strengths of the three studies included in this scoping review: (1) the measurement instruments involve simplified and objective questions, contributing to their understanding and facilitating the response; (2) adequate values of the psychometric analysis validation process, thus ensuring valid and accurate instruments to assess the adherence of people living with HIV to antiretroviral therapy in Brazil; and (3) situational diagnostic capacity regarding therapeutic adherence. The instruments enable continuous monitoring, contributing to decision-making together with the patient towards their effective adherence to the ART.

However, the instruments also present some weaknesses. As for the CEAT-HIV^28^, sample size bias is its weak point. In this study, we could identify whether the sample size considered is ideal for studies involving instrument validation. It is worth remembering that the established cutoff point should be used with caution as the questionnaire is a self-administered instrument. The proposed validation and reproducibility of a self-efficacy scale for adherence of children and adolescents to the ART^29^ considered the responses of parents or caregivers rather than the perception of the target study population. This is so far the only instrument validated in Brazil that assesses adherence of children and adolescents to antiretroviral therapy.

The WebAd-Q^30^ instrument, unlike the other two studies included, considers only the patient-related determinant associated with adherence. Finally, as these are self-administered instruments, they are subject to the Hawthorne’s effect, i.e., respondents may be influenced in their answers when considering the presence and/or possible “judgment” of the professional who will receive the answers regarding adherence to the ART. We highlight the fact that the studies were validated in people living with HIV in Brazil; however, the scope in the use of the questionnaires should be understood with caution, as instruments were validated considering the reality of a specific region or service in the country. In this sense, one should verify the feasibility of using the instruments in the scope of a national study, seeking to reflect the heterogeneity of both people under treatment in the country, and the traits of the services that assist them.

Even with the intrinsic limitations of this review, especially considering the large volume of studies that used different instruments to measure adherence of patients to antiretroviral therapy in Brazil, only three studies were validated from psychometric analyses for the national context. Thus, the quantity and methodological specificities of each study identified make it impossible to compare the realities and contexts in which instruments were validated. However, it reinforces the need for additional investments; that is, being (re)used in different settings. Furthermore, we believe that the results of this paper depict the scientific literature in this time and space, considering the consultation of the six main databases for the compilation of instruments to measure adherence to ART, validated in Brazil. For safety purposes, searches on the Preprints and gray literature servers were included.

We emphasize that this scoping review could gather and present, in a simplified and accessible way, the instruments validated in Brazil that assess adherence of people living with HIV to antiretroviral therapy. The studies included in this review enable the assessment of adherence of children, adolescents and adults living with HIV. In this sense, health professionals working in the care of these people can frequently identify and monitor their adherence and propose specific strategies for each person, in order to consolidate good adherence and intervene in the treatment of those not engaged in therapy. In summary, the use of validated instruments to measure adherence to long-term treatment can support public measures, as well as determine the influence or not of the determinants that act negatively on adherence, as established by the World Health Organization.

## CONCLUSION

The instruments to assess adherence to antiretroviral therapy for people living with HIV included in this scoping review are strategies validated for the Brazilian context. However, their (re)use in different settings and contexts of the nation should be expanded. The use of these instruments by health professionals can improve the understanding of factors that act negatively or positively on adherence to ART, and the proposition of strategies intended to consolidate good adherence and intervene in the treatment of patients with low therapeutic engagement. We emphasize the need for further studies to develop an instrument for national use, which has its validation process in more cities and regions of Brazil.
