# Supplementary material for: Instruments to assess adherence to medication in people living with HIV: a scoping review
Source: Rev Saude Publica. 2022 Nov 18;56:112. doi: 10.11606/s1518-8787.2022056004475 (PMC9749734; doi:10.11606/s1518-8787.2022056004475)
Supplement: Translation

# Translation: Instrumentos para avaliar a adesão medicamentosa em pessoas vivendo com HIV: uma revisão de escopo

**Authors:** André Pereira dos Santos, Jéssica Fernanda Corrêa Cordeiro, Isabela Fernanda Larios Fracarolli, Euripedes Barsanulfo Gonçalves Gomide, Denise de Andrade

**Affiliations:** I Brasil Universidade de São Paulo. Escola de Enfermagem de Ribeirão Preto. Ribeirão Preto, SP, Brasil; II Brasil Universidade de São Paulo. Escola de Educação Física e Esporte de Ribeirão Preto. Grupo de Estudos e Pesquisa em Antropometria, Treinamento e Esporte. Ribeirão Preto, SP, Brasil; III Brasil Universidade de São Paulo. Escola de Educação Física e Esporte de Ribeirão Preto. Ribeirão Preto, SP, Brasil; IV Brasil Human Exposome and Infectious Diseases Network. Ribeirão Preto, SP, Brasil; V Brasil Claretiano – Centro Universitário. Batatais, SP, Brasil

**Keywords:** Infecções por HIV, terapia, Adesão à Medicação, Recusa do Paciente ao Tratamento, Sobreviventes de Longo Prazo ao HIV, Revisão

## Abstract

**OBJETIVO:**

Compilar os instrumentos validados no Brasil para avaliação da adesão de pessoas vivendo com HIV à terapia antirretroviral.

**MÉTODOS:**

Revisão de escopo, utilizando as bases de dados Web of Science, Scopus, Medline (via PubMed), Embase, BDENF, CINAHL e Lilacs. Em complementação, os servidores Preprints bioRxiv, Google Scholar e OpenGrey foram verificados. Para a busca, não houve restrição de idioma e considerou artigos publicados a partir do ano de 1996.

**RESULTADOS:**

Três publicações foram incluídas na síntese qualitativa. Os instrumentos identificados foram o “Questionário para Avaliação da Adesão ao Tratamento Antirretroviral”, desenvolvido em Porto Alegre (RS) e publicado em 2007; a “Escala de autoeficácia para adesão ao tratamento antirretroviral em crianças e adolescentes com HIV/Aids”, desenvolvida em São Paulo (SP) e publicada em 2008; e o “WebAd-Q, um instrumento de autorrelato para monitorar a adesão à terapia antirretroviral em serviços de HIV/Aids no Brasil”, desenvolvido em São Bernardo do Campo (SP) e publicado em 2018. Os instrumentos foram validados no Brasil e apresentaram valores estatisticamente aceitáveis para as qualidades psicométricas.

**CONCLUSÃO:**

Os instrumentos para avaliar a adesão de pessoas vivendo com HIV à terapia antirretroviral são estratégias validadas para o contexto do Brasil. Todavia há que se expandir a (re)utilização em diferentes cenários e contextos da nação. A utilização desses instrumentos por profissionais da saúde pode melhorar a compreensão dos fatores que atuam negativa e positivamente na adesão à terapia antirretroviral, e a proposição de estratégias com o objetivo de consolidar a boa adesão e intervir no tratamento das pessoas com baixo engajamento terapêutico.

## INTRODUÇÃO

Com o advento da terapia antirretroviral (TARV), após 1987, em alguns lugares do mundo, e no Brasil a partir de 1996, com o acesso público, gratuito e universal, os avanços na constituição e na estratégia de administração dos antirretrovirais transformou mundialmente a compreensão da infecção pelo *Human immunodeficiency virus* (HIV), associado ou não a *acquired immunodeficiency syndrome* (Aids), de uma doença fatal, para uma infecção crônica^1,2^.

Mesmo com a remissão prolongada do HIV descrita na literatura^3,4^, até o momento, não há cura e o tratamento que leva a carga viral a nível indetectável (< 40 cópias/ml), tem como principal componente a adesão do paciente à terapia antirretroviral^5^. O controle do HIV e a manutenção da saúde são promovidos pelo uso contínuo e adequado da TARV, cuja efetividade está atrelada à ingestão dos medicamentos conforme a prescrição, determinando sua adesão ao tratamento. Para uma adesão satisfatória, a utilização dos medicamentos antirretrovirais deve ser a mais próxima possível da prescrição fornecida pela equipe de saúde, de forma a cumprir horários, doses e demais indicações^6^. Altos níveis de adesão têm sido consistentemente associados a melhores resultados virológicos, imunológicos e clínicos, com consequente aumento na sobrevida e qualidade de vida de pessoas vivendo com HIV^8^. Adicionalmente, considerando que um dos pilares dos programas de controle da epidemia de HIV no mundo é a estratégia denominada I = I (indetectável = intransmissível), a adesão à terapia impacta positivamente na redução do número de transmissões de parceiros vinculados^9,10^. Por outro lado, o abandono do tratamento medicamentoso ou a adesão incorreta podem facilitar o contágio por doenças oportunistas e levar à morte. Em suma, a não adesão à terapia antirretroviral impacta negativamente em aspectos sociais e políticos, tanto pelo investimento público realizado quanto pelo controle da epidemia^11^.

Nos últimos anos, o Brasil registra uma diminuição no número de infecções pelo HIV. Entre os anos de 2007 e 2020, foram notificados no Sistema de Informação de Agravos de Notificação 342.459 casos de infecção pelo HIV^11^. O Sistema Único de Saúde manteve suas ações para garantir o diagnóstico e tratamento para o HIV, mesmo durante a pandemia da covid-19. O Ministério da Saúde elaborou um painel de “Monitoramento durante a pandemia da covid-19 – dados relacionados ao HIV”, em que apresentou informações importantes sobre os números de pessoas que iniciaram a terapia antirretroviral a cada ano, por exemplo, em 2019, um total de 68.482 pacientes aderiram à TARV; já no ano seguinte, em 2020, foram 55.180 novas adesões^12^.

As barreiras e os facilitadores para a adesão à terapia antirretroviral são multifatoriais, envolvendo questões sociais, econômicas, sistêmicas/profissionais (equipes de saúde), efeitos colaterais ao tratamento, à doença, e as particularidades da pessoa vivendo com HIV^13^. Cada país e suas regiões podem apresentar especificidades que se configuram como barreiras ao tratamento; por exemplo, países como Uganda, Tanzania e Botswana podem apresentar obstáculos em que a fome, o tempo de espera pelo medicamento e os custos de transporte são fortes complicadores para a adesão à TARV^14^. No Brasil, os principais fatores associados a não adesão a essa terapia são os aspectos sociodemográficos, como a idade (jovem), estado civil (solteiro), cor da pele autorreferida (não branca), escolaridade (baixo nível), renda (baixa condição socioeconômica) e a vulnerabilidade ao HIV/Aids, como o uso de drogas lícitas e ilícitas, uso dos serviços de saúde (não adesão às consultas e contato com mais de um serviço de saúde), e o acompanhamento clínico laboratorial (percepção dos efeitos colaterais e complexidade do regime terapêutico)^15,16^.

Devido aos diferentes instrumentos de medidas adotados regionalmente, não existe uma estimativa nacional de adesão à terapia antirretroviral, contudo, uma recente revisão de literatura observou que essa taxa varia entre 18% e 74,3%, dependendo da localidade^17^.

Dentre os diferentes instrumentos e técnicas utilizados para avaliar a adesão da pessoa vivendo com HIV à terapia estão o registro de dispensação dos antirretrovirais^18^, a análise do prontuário médico^19^, questionário de autopreenchimento^20^, o questionário simplificado para avaliação da adesão à TARV-SMAQ^21^ etc. Contudo, observa-se que as propostas para essa avaliação em serviços de saúde no Brasil não utilizam instrumentos e técnicas validados^17^, o que pode levar a erro de verificação e interpretação dos resultados^22^.

Assim, o presente estudo teve como objetivo mapear os instrumentos validados no Brasil para avaliar a adesão de pessoas vivendo com HIV à terapia antirretroviral.

## MÉTODOS

### Delineamento do Estudo

Estudo de revisão do tipo *scoping review* ou revisão de escopo, com a intenção de abordar assuntos amplos, com foco em resultados mais abrangentes. Esse tipo de revisão identifica, examina e sistematiza rigorosamente e efetivamente um conceito ou características singulares de cada estudo ao identificar a natureza de um amplo campo do conhecimento^23,24^. A revisão de escopo é sistematizada e para sua realização, algumas etapas são exigidas. Dentre essas etapas, cinco estágios são considerados obrigatórios e um opcional: (1) identificação da questão de pesquisa; (2) identificação dos estudos relevantes; (3) seleção dos estudos; (4) mapeamento dos dados; (5) agrupamento, análise e resumo dos dados; e (6) consulta a pesquisadores (opcional)^23,24^. Esta revisão de escopo seguiu as diretrizes da lista de conferências de itens para revisões sistemáticas e extensão de metanálises para revisões de escopo (Prisma-ScR)^25^.

### Definição da Pergunta

Ao determinar a pergunta de investigação, optou-se pela utilização da estratégia *population (P), concept (C) and context (C)*^26^. Em que, (P) – pessoas vivendo com HIV que fazem uso de terapia antirretroviral, (C) instrumentos de adesão à terapia antirretroviral, e (C) instrumentos validados no Brasil. Assim, a questão norteadora deste estudo foi: Há instrumentos validados no Brasil para avaliar a adesão à terapia antirretroviral em pessoas vivendo com HIV?

### Período

A busca dos estudos foi realizada em dezembro de 2020 por dois pesquisadores, de forma independente, evitando o viés no número de artigos.

### Coleta de Dados

As principais bases de dados da área da saúde foram selecionadas para busca: *Web of Science* (WOS/ISI), Scopus, *Medical Literature Analysis and Retrieval Online* (Medline/PubMed), Embase, BDENF, *The Cumulative Index to Nursing and Allied Health Literature* (CINAHL) e Literatura Latino-Americana e do Caribe em Ciências da Saúde (Lilacs). Em complementação, os servidores *Preprints bioRxiv, Google Scholar* e *OpenGrey* foram verificados por apresentarem reconhecimento na comunidade acadêmica e devido ao expressivo número de documentos disponíveis.

### Critérios de Seleção

Foram inseridos nesta pesquisa estudos primários, descritivos, revisões, editoriais e manuais publicados a partir de 1996 (início da implantação da terapia antirretroviral no Brasil como política de acesso público e universal contra o HIV), e sem restrição de idioma. Foram selecionados os textos completos disponíveis e que respondessem à pergunta de investigação. Artigos repetidos em mais de uma fonte de dados foram contabilizados apenas uma vez. Todos os estudos encontrados na busca foram importados para o *software* Rayyan^®^, no qual foi realizado todo o processo de análise e seleção.

### Instrumento Utilizado para a Coleta das Informações

Os descritores utilizados nesta pesquisa foram selecionados por meio dos bancos de dados “Descritores em Ciências da Saúde” (DeCS) e “*Medical Subject Head Medical Subject Headings*” (MESH), que estão destacados no Quadro 1.

**Quadro 1:** Estratégias de buscas utilizadas nas bases de dados e repositórios

Base de dados	Estratégia de busca
PubMed/Medline	(“Acquired Immunodeficiency Syndrome” OR HIV) AND (“medication adherence” OR compliance) AND (“Surveys and Questionnaires”)
CINAHL	(“Human Immunodeficiency Virus” OR “Anti-HIV Agents”) AND (“Medication Compliance”) AND (“Structured Questionnaires” OR “Surveys”)
Web of Science	(Acquired Immunodeficiency Syndrome OR HIV) AND (medication adherence OR compliance) AND (Surveys and Questionnaires)
Scopus	(“Acquired Immunodeficiency Syndrome” OR HIV) AND (“medication adherence” OR compliance) AND (“Surveys and Questionnaires”)
Embase	Acquired Immunodeficiency Syndrome AND medication compliance AND questionnaire
BVS (Lilacs, BDENF)	(Acquired Immunodeficiency Syndrome OR HIV) AND (medication adherence OR compliance) AND (Surveys and Questionnaires)
	(Síndrome de inmunodeficiencia adquirida O VIH) Y (adherencia o cumplimiento a la medicación) Y (Encuestas y cuestionarios)
BioRxiv	“Acquired Immunodeficiency Syndrome AND medication adherence AND Surveys and Questionnaires”
Google Scholar	“Acquired Immunodeficiency Syndrome AND medication adherence AND Surveys and Questionnaires”
OpenGrey	“Acquired Immunodeficiency Syndrome AND medication adherence AND Surveys and Questionnaires”

### Tratamento e Análise dos Dados

Na sequência, realizou-se o agrupamento, análise e resumo dos dados extraídos por dois revisores independentes. As dúvidas e incongruências foram analisadas e discutidas por um terceiro revisor. Os dados foram registrados em planilha Microsoft Office Excel^®^, versão 2010, e apresentados quanto à identificação (autoria e ano), objetivo, método, principais resultados, vieses e limitações do estudo^27^.

## RESULTADOS

A busca na literatura científica por esses instrumentos validados que avaliam a adesão dos pacientes à terapia antirretroviral no Brasil resultou na inclusão de três estudos. Na Figura observa-se o processo de busca, identificação, exclusão e seleção dos estudos, de acordo com as recomendações do Prisma.

**Figura:** Fluxograma da seleção dos estudos recuperados nas bases de dados, adaptado do *Preferred Reporting Items for Systematic Review and Meta-Analyses* (Prisma), sobre os instrumentos validados que avaliam a adesão à terapia antirretroviral em pessoas vivendo com HIV no Brasil.

Os instrumentos identificados foram o “Questionário para Avaliação da Adesão ao Tratamento Antirretroviral”, desenvolvido em Porto Alegre e publicado em 2007^28^, a “Escala de autoeficácia para adesão ao tratamento antirretroviral em crianças e adolescentes com HIV/Aids”, desenvolvida em São Paulo e publicada em 2008^29^, e o “WebAd-Q, um instrumento de autorrelato para monitorar a adesão à terapia antirretroviral em serviços de HIV/Aids no Brasil”, desenvolvido em São Bernardo do Campo e publicado em 2018^30^. Todas as publicações incluídas na síntese qualitativa desta revisão de escopo foram escritas no idioma português e inglês, exceto a publicação de Remor; Milner-Moskovics; Preussler, 2007^28^, publicada exclusivamente no idioma português. As três publicações incluídas estavam presentes na base de dados Scopus. As informações extraídas desses estudos sobre identificação, objetivo, método, resultados principais e vieses/limitações estão apresentadas no Quadro 2.

**Quadro 2:** Síntese dos estudos incluídos na revisão de escopo relativos à pergunta: “Há instrumentos validados no Brasil para avaliar a adesão à terapia antirretroviral em pessoas vivendo com HIV?”

Identificação (autoria/ano)	Objetivo	Método	Principais resultados	Limitações do estudo
Remor E, Milner-Moskovics J, Preussler G., 2007^28^	Traduzir, adaptar e validar o questionário para seu uso no Brasil: *Cuestionario para la Evaluación de la Adhesión al Tratamiento Antiretroviral (CEAT-VIH)*”. Trata-se de um instrumento autoaplicável para a identificação do grau de adesão ao tratamento antirretroviral em pacientes com infecção pelo HIV.	Estudo metodológico subsidiado em um questionário traduzido do original em espanhol ao português, processo de retradução (espanhol/português/espanhol), com avaliação verbal da compreensão com um pequeno grupo de pacientes. A análise das propriedades psicométricas envolveu 59 pacientes em tratamento antirretroviral de Porto Alegre/ Rio Grande do Sul, Brasil. A consistência interna e a, validez relacionada a um critério externo, sensibilidade e especificidade subsidiaram a validação. A versão final do CEAT-VIH (20 perguntas), a pontuação total é obtida pela soma de todos os itens (valor mínimo possível 17, valor máximo possível 89).	Foi observada uma adequada confiabilidade do questionário (α = 0,64) e validade relacionada a um critério externo (carga viral; r = -0,48; p < 0,001). Também, se observou adequada sensibilidade (79,2%) e especificidade (57,1%) do questionário para a detecção entre indivíduos com carga viral indetectável e detectável. O ponto de corte ótimo sugerido pela análise é de ≥ 76. Valores abaixo indicam insuficiente adesão ao tratamento e associação a uma carga viral detectável. A esta pontuação se associa uma sensibilidade de 79,2% e especificidade de 57,1%.	Não é apresentado o cálculo para o tamanho amostral. Estudo em apenas uma única instituição dificultando a generalização; portanto, não é um estudo representativo Nacional. O ponto de corte estabelecido deve ser utilizado com cautela pelo fato de o questionário ser um instrumento autoaplicável. Efeito Hawthorne.
Costa LS, Latorre M do RD de O, Silva MH da, Bertolini DV, Machado DM, Pimentel SR, et al., 2008^29^	Validar uma escala de autoeficácia para adesão ao tratamento antirretroviral em crianças e adolescentes com HIV/Aids, levando em consideração a perspectiva dos pais/responsáveis, e avaliar a sua reprodutibilidade.	Estudo metodológico realizado no Hospital-Dia do Centro de Referência e Treinamento em DST/Aids de São Paulo. Foram entrevistados os pais/responsáveis de 54 crianças e adolescentes de 6 meses a 20 anos que passaram em consulta de rotina pelo serviço. Os dados de autoeficácia da adesão à prescrição antirretroviral, foi calculada de duas maneiras: análise fatorial e fórmula já definida. A consistência interna da escala foi verificada pelo coeficiente α de Cronbach. A validade foi avaliada pela comparação das médias dos escores entre grupos de pacientes aderentes e não aderentes ao tratamento antirretroviral (teste de Mann-Whitney) e cálculo do coeficiente de correlação de Spearman entre os escores e parâmetros clínicos. A reprodutibilidade foi verificada por meio do teste de Wilcoxon, pelo coeficiente de correlação intraclasse e através dos Plots de Bland-Altman.	A escala de autoeficácia é constituída de 21 questões. Apresentou boa consistência interna (α = 0,87) e boa reprodutibilidade (CCI = 0,69 e CCI = 0,75). Quanto à validade, a escala de autoeficácia para seguir prescrição antirretroviral conseguiu discriminar pacientes com adesão e com adesão insuficiente ao tratamento antirretroviral (p = 0,002) e apresentou correlação significativa com a contagem de CD4 (r = 0,28; p = 0,04). Adicionam-se aos resultados que as crianças/adolescentes com adesão ao tratamento antirretroviral têm maior expectativa de autoeficácia do que aqueles com adesão insuficiente. A escala de autoeficácia para seguir prescrição antirretroviral pode ser utilizada para avaliar a adesão à terapia antirretroviral em crianças e adolescentes com HIV/Aids, levando em consideração a perspectiva dos pais/cuidadores.	Não é um estudo representativo Nacional. Foram os pais/cuidadores que responderam pelas crianças/adolescentes. Não foi considerada a percepção da população alvo do estudo. Efeito Hawthorne.
Vale FC, Santa-Helena ET de, Santos MA, Carvalho WM do ES, Menezes PR, Basso CR, et al., 2018^30^	Apresentar o desenvolvimento e a validação do Questionário WebAd-Q, um instrumento de autorrelato para monitorar a adesão à terapia antirretroviral em serviços de HIV/Aids no Brasil.	Estudo metodológico. O O WebAd-Q é um questionário eletrônico que contém três perguntas sobre a tomada dos antirretrovirais na última semana. Foi construído a partir de entrevistas e grupos focais com 38 pacientes. Sua validade foi verificada em estudo com uma amostra de 90 pacientes maiores de 18 anos, sob terapia antirretroviral há pelo menos três meses. Foram utilizadas as seguintes medidas de adesão comparativas: monitoramento eletrônico, contagem de pílulas e entrevista de autorrelato. O WebAd-Q foi respondido no sexagésimo dia por duas vezes, com intervalo mínimo de uma hora. A carga viral dos pacientes foi obtida nos registros do serviço. Foi analisada a concordância entre as respostas ao WebAd-Q, associações e correlações com a carga viral e o desempenho em comparação às demais medidas de adesão.	Entre os pacientes convidados, 74 (82,2%) responderam ao WebAd-Q. Não foram relatadas dificuldades em responder ao questionário. O tempo médio de resposta foi de 5 min 47 seg. O conjunto das três questões do WebAd-Q obteve concordância de 89,8%, com Kappa de 0,77 (IC95% 0,61–0,94). As respostas de adesão insuficiente do WebAd-Q associaram-se à carga viral detectável. Foram obtidas correlações moderadas da carga viral com escala de adesão insuficiente segundo o WebAd-Q. Para as três perguntas do WebAd-Q, pacientes com respostas de adesão insuficiente foram também apontados como menos aderentes segundo as demais medidas de adesão. O WebAd-Q atendeu aos principais requesitos de validação de questionários, apresentou elevada compreensão dos participantes e, associação com a carga viral, bem como obteve concordância e bom desempenho em comparação a medidas concorrentes.	Não é um estudo representativo Nacional. Perda amostral que pode ter reduzido o poder estatístico do estudo. Efeito Hawthorne.

Em síntese, todos os estudos incluídos nesta revisão validaram seus instrumentos, indicando valores estatisticamente aceitáveis no que tange às qualidades psicométricas de cada instrumento. O principal viés/limitação observado foi a especificidade da amostra, ou seja, sugerem cautela no uso dos instrumentos ao verificar a adesão à terapia por pessoas de outras regiões do Brasil.

## DISCUSSÃO

Realizar o mapeamento dos instrumentos de medida da adesão à terapia antirretroviral por pessoas vivendo com HIV, validados no Brasil, norteou esta revisão de escopo. Até onde sabemos, este é o primeiro estudo que reuniu as estratégias passíveis de utilização no Brasil. A identificação desses instrumentos é o principal achado do nosso estudo, que poderá orientar a produção de conhecimento relevante para o planejamento e gestão do serviço de saúde. Esta revisão avança ainda no campo da adesão à TARV ao analisar e compilar os instrumentos, que a partir dos resultados obtidos, podem ser interpretados de forma precisa e segura, a luz do rigor psicométrico de cada proposta.

Considerando o impacto positivo na qualidade de vida das pessoas vivendo com HIV que apresentam adequada adesão à terapia antirretroviral, bem como os prejuízos da irregularidade ou descontinuação do tratamento, é importante destacar que a adesão é um processo colaborativo vinculado ao princípio da autonomia que implica na participação ativa do paciente no cuidado com sua saúde. Essa cooperação entre a pessoa vivendo com HIV e a equipe multiprofissional promove a aceitação e a incorporação do esquema terapêutico no cotidiano de seu tratamento^31^.

Em estudos envolvendo a saúde pública no Brasil, os instrumentos apresentados nesta revisão^28^podem melhorar a compreensão da prevalência, incidência e fatores de risco para a adesão insuficiente à TARV, bem como proposição de estratégias para melhorar à adesão medicamentosa. Além disso, a utilização de instrumentos validados contribuirá para intervenções de profissionais da saúde no cuidado e melhores resultados sobre o bem-estar geral das pessoas vivendo com HIV. As perguntas dos instrumentos incluídos em nossa revisão contemplam os cinco determinantes que atuam negativa ou positivamente na adesão a tratamentos de longa duração estabelecidos pela Organização Mundial da Saúde: fatores sociais e econômicos, relacionados ao sistema/equipe de saúde, ao tratamento, à doença, e ao paciente^13^.

O “Cuestionario para la Evaluación de la Adhesión al Tratamiento Antirretroviral” (CEAT-VIH), construído em 2002^32^, é um instrumento simplificado que contempla 20 perguntas e aplicável a pessoas adultas vivendo com o HIV. Além de sua validação no Brasil^28^, o CEAT-VIH foi validado no Canadá^33^, Portugal^34^ e Peru^35^, indicando sua utilidade e validade para avaliar a adesão à terapia antirretroviral em diferentes contextos. No estudo de validação no Brasil, foi observado uma associação significativa e positiva entre o número de comprimidos e a carga viral (r = 0,32; p = 0,01), indicando que a quantidade de comprimidos da terapia pode ser um aspecto dificultador para a adesão ao tratamento. A administração de um número ≤ 10 comprimidos está associada à melhor adesão. Observou-se ainda associação inversa significativa entre o grau de adesão à terapia antirretroviral e a carga viral, (r = -0,48; p = 0,000). O ponto de corte ótimo sugerido pela análise é de ≥ 76. Valores abaixo indicam adesão insuficiente ao tratamento e associação a uma carga viral detectável. A carga viral é o indicador clínico mais adotado em estudos que avaliam a precisão de instrumentos para avaliar a adesão à TARV, indicando a relevância da adesão medicamentosa para um melhor prognóstico de recuperação da doença^17,36^. A maioria dos itens do CEAT-VIH cumpriu os critérios de qualidade esperados e não houve valores perdidos, o que indica que todas as perguntas foram compreendidas pelos pacientes e puderam ser respondidas.

Alguns estudos nacionais utilizaram o CEAT-VIH, como o desenvolvido na cidade de Fortaleza (CE)^37^, outro na região centro-oeste do estado do Rio Grande do Sul^38^, e em cinco serviços de assistência especializada no estado de Pernambuco^39^. Nesses diferentes contextos, observou-se baixa adesão à terapia antirretroviral. Uma realidade que legitima o uso do CEAT-VIH enquanto ferramenta a ser utilizada pelos profissionais da saúde no planejamento do cuidado e intervenção nas situações que interferem na adesão.

Até o presente momento, o estudo de validação e reprodutibilidade de uma escala de autoeficácia para adesão à TARV em pais ou cuidadores de crianças e adolescentes vivendo com HIV/Aids é o primeiro no contexto brasileiro. A boa consistência interna da escala na amostra total de crianças e adolescentes (α = 0,87) evidenciou alta associação entre os itens da escala, sugerindo que ela é confiável para medir autoeficácia em crianças e adolescentes com HIV/Aids, considerando a perspectiva dos pais/cuidadores. Também foi observado que as crianças/adolescentes aderentes à terapia antirretroviral têm maior expectativa de autoeficácia do que aqueles com adesão insuficiente, indicando que uma maior autoeficácia está associada a melhores respostas dos linfócitos TCD 4+ (r = 0,28; p = 0,040). É sugerido no artigo de validação que na rotina clínica seja utilizado o escore 2 de autoeficácia, pois é calculado por uma fórmula pré-definida e facilmente aplicável. Já o escore 1 – também apresentado no artigo – é um escore composto pelas questões que mais se correlacionaram com as perguntas da própria escala, sendo necessário que o pesquisador tenha familiaridade com análise fatorial, além do uso de um pacote estatístico.

Alguns estudos nacionais utilizaram o escore 2 da escala de autoeficácia para avaliar a adesão de crianças e adolescentes à terapia antirretroviral, dentre eles um estudo realizado em Brasilia-DF^40^e outro na cidade de São Paulo^41^. No estudo publicado em 2010^40^, observaram-se dificuldades relacionadas com a ingestão de medicamentos fora do ambiente doméstico, a perda de doses, e com atrasos na ingestão do medicamento. Já no estudo publicado em 2012^41^, foi identificada alta taxa de adesão à TARV, independentemente do perfil sociodemográfico dos pais/cuidadores. A escala de autoeficácia para avaliar a adesão de crianças e adolescentes à terapia medicamentosa é constituída por 21 questões. Essa abrangência de informações possibilita um detalhamento maior dos aspectos dificultadores para a não adesão, bem como aqueles que facilitam a adesão terapêutica.

O instrumento WebAd-Q é um questionário autoaplicável desenvolvido em serviços de atenção à pessoa vivendo com HIV. No estudo de desenvolvimento e validação^30^ foi observado alto nível de concordância teste-reteste e boa concordância entre as respostas e outros indicadores de adesão. Em semelhança aos dois outros instrumentos incluídos nesta revisão de escopo^28,29^, a escala de adesão insuficiente obtida pelo WebAd-Q apresentou associação moderada quando comparada ao desfecho clínico (carga viral), ou seja, quanto menor a adesão à terapia antirretroviral, maior a carga viral verificada. O WebAd-Q contempla apenas a dimensão paciente dentro dos cinco determinantes associados à adesão a tratamentos de longa duração estabelecidos pela Organização Mundial da Saúde^13^. Ele avalia a tomada de todos os medicamentos nos horários e doses prescritas durante a última semana, com boa fidedignidade.

O WebAd-Q foi utilizado para avaliar a adesão à TARV em um *Survey* de amostra não representativa, realizado nas cinco regiões do Brasil^42^. Foi observada alta proporção de não adesão à terapia antirretroviral. Os autores mencionam que o WebAd-Q é um instrumento de rápida aplicação, o que favorece na rotina de atendimento à pessoa vivendo como HIV, possibilitando a identificação de lacunas no fornecimento de aconselhamento e orientação sobre a terapia e permite, ainda, o desenvolvimento de estratégias inovadoras para prevenir a não adesão.

Destacamos os seguintes pontos fortes dos três estudos incluídos nesta revisão de escopo: (1) os instrumentos de medida envolvem perguntas simplificadas e objetivas, contribuindo para sua compreensão e facilitação da resposta; (2) Valores adequados do processo de validação da análise psicométrica, garantindo, assim, instrumentos validos e precisos para avaliarem a adesão de pessoas vivendo com HIV à terapia antirretroviral no Brasil; e (3) Capacidade de diagnóstico situacional referente à adesão terapêutica; os instrumentos possibilitam o monitoramento contínuo, contribuindo para a tomada de decisão junto com o paciente em prol da sua efetiva adesão à TARV.

Contudo os instrumentos apresentam também algumas fragilidades. No caso do CEAT-VIH^28^, o viés do tamanho amostral se apresenta como seu ponto fraco. Neste trabalho, não foi possível identificar se o tamanho amostral considerado é o ideal para estudos que envolvem validação de instrumentos. É importante lembrar que o ponto de corte estabelecido deve ser utilizado com cautela, devido ao fato de o questionário ser um instrumento autoaplicável. A proposta de validação e reprodutibilidade de uma escala de autoeficácia para adesão de crianças e adolescentes à TARV^29^ considerou as respostas de pais ou cuidadores, e não a percepção da população alvo do estudo. Contudo, esse é até o momento o único instrumento validado no Brasil que avalia a adesão de crianças e adolescentes à terapia antirretroviral.

O instrumento WebAd-Q^30^, diferente dos outros dois estudos incluídos, considera apenas o determinante associado à adesão relacionado ao paciente. Por fim, por serem instrumentos autoaplicáveis, pode haver o efeito Hawthorne, ou seja, os respondentes podem ser influenciados em suas respostas ao considerar a presença e/ou possível “julgamento” do profissional que receberá as respostas referentes à adesão à TARV. Salienta-se o fato de os estudos serem validados em pessoas vivendo com HIV do Brasil, contudo, a abrangência no uso dos questionários deve ser compreendida com cautela, pois os instrumentos foram validados considerando a realidade de uma região ou serviço específico da nação. Nesse sentido, é necessário verificar a viabilidade no uso dos instrumentos no escopo de um estudo nacional, buscando refletir a heterogeneidade, tanto das pessoas em tratamento no país quanto das características dos serviços que as assistem.

Mesmo diante das limitações intrínsecas desta revisão, especialmente, considerando o elevado volume de trabalhos que utilizaram diferentes instrumentos de medida da adesão de pacientes à terapia antirretroviral no Brasil, apenas três foram validados a partir de análises psicométricas para o contexto nacional. Assim, a quantidade e as especificidades metodológicas de cada estudo identificado inviabilizam a comparação das realidades e contextos em que os instrumentos foram validados. Todavia, reforça a necessidade de investimentos adicionais; ou seja, na (re)utilização em diferentes cenários. Além disso, acreditamos que os resultados deste trabalho expressam um retrato da literatura científica nesse tempo e espaço, considerando a consulta às seis principais bases de dados para a compilação dos instrumentos de medida da adesão à TARV, validados no Brasil. Por segurança, foram incluídas as pesquisas nos servidores *Preprints* e na literatura cinza.

Destacamos que esta revisão de escopo foi capaz de reunir e apresentar, de forma simplificada e acessível, quais são os instrumentos validados no Brasil que avaliam a adesão de pessoas vivendo com HIV à terapia antirretroviral. Os estudos incluídos nesta revisão possibilitam a avaliação da adesão de crianças, adolescentes e adultos vivendo com HIV. Nesse sentido, os profissionais da saúde que atuam no cuidado a essas pessoas podem identificar e monitorar frequentemente a sua adesão e propor estratégias específicas para cada pessoa, no intuito de consolidar a boa adesão e intervir no tratamento daqueles não engajados à terapia. Em síntese, a utilização de instrumentos validados para medir a adesão a tratamentos de longa duração pode subsidiar a ação de medidas públicas, bem como, determinar a influência ou não dos determinantes que atuam negativamente na adesão, conforme estabelecido pela Organização Mundial da Saúde.

## CONCLUSÃO

Os instrumentos para avaliar a adesão de pessoas vivendo com HIV à terapia antirretroviral incluídos nesta revisão de escopo são estratégias validadas para o contexto brasileiro. Todavia há que se expandir a (re)utilização em diferentes cenários e contextos da nação. A utilização desses instrumentos por profissionais da saúde, pode melhorar a compreensão dos fatores que atuam negativa ou positivamente na adesão à TARV, e a proposição de estratégias com o objetivo de consolidar a boa adesão e intervir no tratamento de pacientes com baixo engajamento terapêutico. Ressalta-se a necessidade de estudos posteriores para o desenvolvimento de um instrumento para uso nacional, que tenha seu processo de validação em mais cidades e regiões do Brasil.
